# Safety and efficacy of the SGLT2 inhibitor dapagliflozin in patients with systemic lupus erythematosus: a phase I/II trial

**DOI:** 10.1136/rmdopen-2022-002686

**Published:** 2022-10-26

**Authors:** Huijing Wang, Ting Li, Fangfang Sun, Zhe Liu, Danting Zhang, Xiangyu Teng, Laurence Morel, Xiaodong Wang, Shuang Ye

**Affiliations:** 1 Department of Rheumatology, Renji Hospital, School of Medicine, Shanghai Jiaotong University, Shanghai, China; 2 Department of Microbiology, Immunology, and Molecular Genetics, University of Texas Health San Antonio, San Antonio, Texas, USA

**Keywords:** lupus erythematosus, systemic, lupus nephritis, outcome and process assessment, health care

## Abstract

**Objective:**

Sodium-glucose cotransporter-2 inhibitors have been identified profound renal/cardiac protective effects in different diseases. Here, we assessed the safety and efficacy of dapagliflozin among adult patients with systemic lupus erythematosus (SLE).

**Methods:**

We conducted a single-arm, open-label, investigator-initiated phase I/II trial of dapagliflozin in Chinese patients with SLE with/without lupus nephritis (LN). Patients received oral dapagliflozin at a daily dose of 10 mg added to the standard of care for 6 months. The primary end point was the safety profile. The secondary efficacy end points were composite assessments of disease activity.

**Results:**

A total of 38 eligible patients were enrolled. Overall, 19 (50%) adverse events (AEs) were recorded, including 8 (21%) AEs leading to drug discontinuation, of which 4 (10.5%) were attributed to dapagliflozin. Two serious AEs (one of major lupus flare and one of fungal pneumonia) were recorded. Lower urinary tract infection was observed in one (2.63%) patient. The secondary end points revealed no improvement of SLE Disease Activity Index scores or proteinuria (among 17 patients with LN); the cumulative lupus flare rate was 18%, and a reduction of ~30% in the prednisone dose was captured. Net changes in body mass index (−0.50 kg/m^2^), systolic blood pressure (−3.94 mm Hg) and haemoglobin levels (+8.26 g/L) were detected. The overall estimated glomerular filtration rate (eGFR) was stable, and there was an improvement in the eGFR slope among patients with LN with a baseline eGFR <90 mL/min/1.73 m^2^.

**Conclusion:**

Dapagliflozin had an acceptable safety profile in adult patients with SLE. Its possible renal/cardiac protective effects and long-term safety issues in patients with SLE, patients with LN in particular, call for further exploration.

**Trial registration number:**

ChiCTR1800015030.

**WHAT IS ALREADY KNOWN ON THIS TOPIC**

- Increased glucose metabolism in immune cells has been reported in patients with systemic lupus erythematosus (SLE), and metabolic modulation approaches have become a hot spot in the management of SLE.
- Sodium-glucose cotransporter-2 inhibitors (SGLT2is) exert profound renal and cardiac protective effects in different human diseases.

**WHAT THIS STUDY ADD**

- This phase I/II trial showed an acceptable safety profile of dapagliflozin add-on therapy in adult patients with SLE.
- No effects in terms of reduced disease activity or proteinuria among patients with LN were detected.

**HOW THIS STUDY MIGHT AFFECT RESEARCH, PRACTICE OR POLICY**

- SGLT2is, as a possible adjunct treatment for renal/cardiac protection in patients with SLE, patients with LN in particular, warrant further properly designed larger-scale, placebo-controlled trials.

## Introduction

Systemic lupus erythematosus (SLE) is a systemic autoimmune disorder characterised by a relapsing course of disease and organ damage accrual.^1^ Recently, taming the overreactive immune system via a metabolic modulation approach has become a hot spot in the management of SLE.^2 3^ Increased glucose metabolism in immune cells has been reported in patients with SLE and mouse models of the disease.^2 4^ The treatment of lupus-prone mice with inhibitors of glycolysis, such as 2-deoxy-D-glucose had beneficial effects.^4^ Repurposing metformin, an old antidiabetic drug, has the potential to reduce the risk of lupus flare in randomised controlled trials (RCTs).^5 6^ A recent crossover study implied that peroxisome proliferator-activated receptor-gamma agonists might decrease cardiovascular risk in patients with SLE.^7^

Dapagliflozin, a sodium-glucose cotransporter-2 inhibitor (SGLT2i), is a new therapy for type 2 diabetes. Its mode of action is to reduce glucose reabsorption in the epithelial cells of the proximal renal tubule of the kidney, which results in decreased blood glucose and glycated haemoglobin levels.^8 9^ Strikingly, four cardiovascular outcome trials demonstrated that treatment with SGLT2is (empagliflozin, canagliflozin and dapagliflozin) in patients with type 2 diabetes had prominent effects on slowing the decline rate of estimated glomerular filtration rate (eGFR) and decreasing albuminuria, as well as a significant reduction in cardiovascular events.^10–13^ Furthermore, the nephroprotective efficacy of SGLT2is was extended to non-diabetic chronic kidney disease (CKD), such as IgA nephropathy.^14 15^ The net gain of SGLT2 inhibition is to reduce renal workload (intraglomerular pressure) and to modulate weight loss and blood pressure (BP).^15–19^ The paradigm for CKD and congestive heart failure management has been shifted accordingly.^20 21^ Interestingly, it has been reported that SGLT2is could block lipopolysaccharide-induced and NLRP3-mediated inflammatory responses and regulate macrophage polarisation via interplay with mammalian target of rapamycin (mTOR) and AMP-activated protein kinase pathways^22 23^; thereby, SGLT2is might further contribute to reducing inflammation, modulating endothelial dysfunction and decelerating atherosclerosis,^21 22^ which are all relevant to the pathophysiology of SLE.

With all the beneficial properties of SGLT2is, they have become appealing candidates for treating patients with SLE, especially those with lupus nephritis (LN).^24^ Recently, a pilot study found an antiproteinuric effect of empagliflozin in five patients with LN.^21^ Despite having a favourable safety profile in patients with different chronic diseases, the safety issue of SGLT2is in patients with SLE has never been properly addressed and should be prioritised in the research agenda. For instance, SGLT2i-induced glycosuria, which potentially creates an environment that is susceptible to bacterial and fungal growth, might increase the risk of urinary tract infections (UTIs),^25 26^ which are not uncommon in patients with SLE.

Here, we initiated this phase I/II trial aiming to investigate the safety and efficacy of dapagliflozin in Chinese adult patients with SLE with or without active LN.

## Methods

### Study design and participants

This study was a single-arm, open-label, investigator-initiated phase I/II trial at the Department of Rheumatology, Renji Hospital, Shanghai Jiao Tong University School of Medicine (Shanghai, China). We enrolled patients aged 18 years and older with confirmed SLE who fulfilled the revised 1997 American College of Rheumatology (ACR) criteria or 2012 Systemic Lupus International Collaborating Clinics classification criteria. Patients with or without active LN (defined as proteinuria level >0.5 g/24 hours at the time of enrolment)^21^ were eligible. Patients with an allergy or intolerance to dapagliflozin or any prior SGLT2i exposure within 1 month before screening were excluded. Other exclusion criteria were as follows: acute infection requiring antibiotics within 1 month before screening; hepatic dysfunction (aspartate aminotransferase or alanine aminotransferase levels >2 times the upper normal limits); an eGFR of <45 mL/min/1.73 m^2^; and current pregnancy or breast feeding. Of note, due to the cumulative evidence that the application of SGLT2is has extended to the non-diabetic population, a diagnosis of diabetes was considered neither an inclusion nor an exclusion criterion.

All patients received stable standard-of-care treatments for at least 1 month before enrolment, when dapagliflozin, at a dose of 10 mg/day, was added-on. During the 6-month trial, the prednisone dose was tapered based on the investigators’ clinical decision. Adjustments of other immunosuppressive agents and antimalarial agents were not allowed. Any increase of these medications or the prednisone dosage was defined as a disease flare as per the Safety of Estrogens in Lupus Erythematosus National Assessment-SLE Disease Activity Flare Index (SFI) definition.^27^ Patients taking renin-angiotensin-aldosterone system inhibitors (RAASis) were also required to be kept on a stable dose, whereas new RAASi treatment was prohibited during the 6-month trial. On the other hand, the tapering of concomitant hypoglycaemic drugs for patients with diabetes was allowed during the trial. Study visits occurred at 2-month intervals, that is, at 0, 2, 4 and 6 months. At each visit, physical examination included body mass index (BMI) and BP measurements; laboratory assessments (blood and urine samples) were collected. Additionally, fasting blood glucose (FBG) and glycated haemoglobin (HbA1c) levels were recorded for patients with a history of diabetes. When a participant had increased symptoms between two study visits, an appointment for an additional visit was scheduled. The trial was registered with www.chictr.org.cn, with clinical trial registration number ChiCTR1800015030.

### Outcome measurements

Outcome assessments were measured at 0, 2, 4 and 6 months. The primary end point was safety and tolerability outcomes as assessed by the occurrence of adverse events (AEs) according to the Medical Dictionary for Regulatory Activities (V.21.0). Of note, glycosuria was not considered an AE.

The secondary efficacy end points were composite assessments of disease activity, including SLE Disease Activity Index 2000 scores; prednisone dosage; proteinuria (for active patients with LN) at each visit compared with baseline; and the frequency of lupus flares, defined by the SFI.

The exploratory efficacy end points were changes in BMI, BP, haemoglobin and eGFR levels at each visit compared with baseline. The 6-month eGFR slope was calculated.^28^

### Statistical analysis

Sample size estimation was indexed by an SGLT2i-related AE with special interest, that is, UTI. The annual incidence of UTI was <5%, according to our previous Met Lupus trial^5^ with patients with SLE. Thus, the enrolment of 40 patients would likely capture no more than one case of UTI for a 6-month trial as a background reference. A modified intention-to-treat (mITT) population, that is, patients who received at least one dose of a treatment, was subjected to assessments for both the safety and efficacy end points. The outcomes were interpreted in the mITT population with the Mann-Whitney U test for categorical variables and two­-tailed Student’s t-test for continuous variables. A p value <0.05 was considered to define significance. Any reported p values in the tests for exploratory end points were considered nominal and should not be interpreted as clinically meaningful. Statistical analyses were carried out with SPSS (V22.0) and GraphPad (V.9.0.0) softwares.

## Results

### Demographics and baseline characteristics

**Figure 1:**
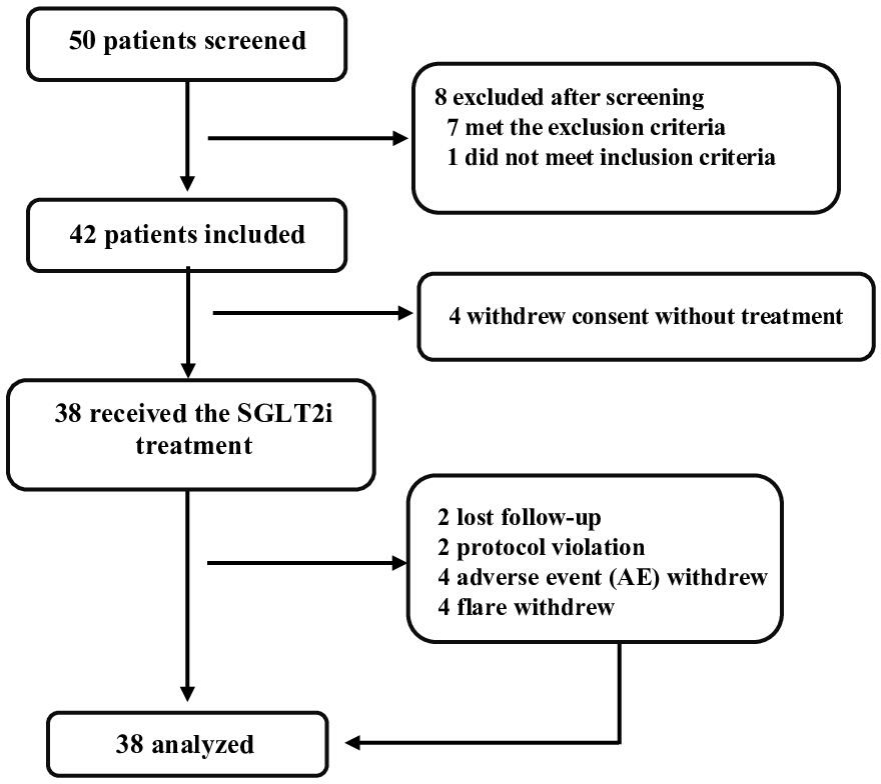
Trial profile. SGLT2i, sodium-glucose cotransporter-2 inhibitor.

The trial screened 50 patients with SLE between 12 June 2017 and 2 May 2018. Eight patients did not meet the eligibility criteria, and four withdrew consent without receiving treatment. The remaining 38 patients who received at least one dose of dapagliflozin treatment were followed up as the mITT population (figure 1). The mean age was 34.51 years (SD 9.99), and 36 (94.74%) patients were women. The mean disease duration was 8.32 years (SD 4.99), and 5 (13.16%) patients had a history of diabetes. They had a baseline SLEDAI score of 4.24 (SD 2.62), of whom, 12 (31.58%) patients had a baseline SLEDAI score ≥6. Among the 17 patients (44.74%) with active LN, 13 (76.47%) were confirmed by renal biopsy. The mean LN disease duration was 2.7 (IQR 2, 6.2) years. Baseline clinical characteristics, including the use of concomitant medications, are shown in table 1.

**Table 1:** Clinical characteristics of the patients with systemic lupus erythematosus (SLE)

Clinical characteristic	All SLE (n=38)	LN (n=17)*	Non-LN (n=21)
Sex, male/female	2/36	1/16	1/20
Age, year	34.51±9.99	33.0±10.03	36.0±10.75
SLE disease duration, year	8.32±4.99	7.33±5.04†	9.25±5.22
History of diabetes‡	5 (13.16%)	3 (17.65%)	2 (9.52%)
Body mass index, kg/m^2^	23.97±3.91	22.93±3.25	24.47±4.44
Blood pressure, mean (SD), mm Hg			
Systolic	121.32±15.33	123.4±14.0	119.68±16.50
Diastolic	82.59±9.63	83.60±8.76	81.79±10.43
SLEDAI, mean±SD	4.24±2.62	5.06±2.46	2.62±1.94
SLEDAI score ≥6	12 (31.58%)	11 (64.71%)	1 (4.76%)
Proteinuria ≥1.5 g/24 hours	11 (28.95%)	11 (64.71%)	
24 hours urine protein (g）		1.7 (1.24, 2.95)	
Haemoglobin	120.24±20.79	114.29±21.0	125.3±19.73
ALT	28.43±13.67	24.43±12.01	30.71±14.80
Serum creatinine	66.74±26.28	73.81±35.14	61.71±15.55
eGFR, mL/min/1.73 m^2^ (EPI)	118.40±27.93	111.66±33.70	123.86±21.57
eGFR <90 mL/min/1.73 m^2^, n (%)	5 (13.16%)	4 (23.53%)	1 (4.76%)
Erythrocyte sedimentation rate	25.03±24.0	28.18±29.00	25.10±19.36
C reactive protein, mg/L	6.20±12.13	3.18±1.80	8.35±15.60
Anti-dsDNA, positive	24 (63.16%)	11 (64.71%)	13 (61.90%)
C3 concentration (g/L）	0.86±0.31	0.88±0.30	0.84±0.32
C3 concentration <0.9 g/L	21 (55.26%)	9 (52.94%)	12 (57.14%)
C4 concentration (g/L)	0.17±0.11	0.20±0.10	0.15±0.11
C4 concentration <0.1 g/L	11 (28.95%)	3 (17.65%)	8 (38.10%)
Treatment			
Prednisone (mg/day）	14.21±7.36	16.32±7.13	12.50±7.25
Prednisone dosage ≥20 mg/day, n (%)	9 (23.68%)	6 (35.29%)	3 (13.64%)
Hydroxychloroquine	33 (86.84%)	15 (88.24%)	18 (85.71%)
RAASis	9 (23.68%)	8 (47.06%)	1 (4.76%)
Other antihypertensive drugs	11 (28.95%)	2 (11.76%)	9 (42.86%)
Immunosuppressive drugs			
Mycophenolate	10 (26.32%)	6 (35.29%)	4 (19.05%)
Azathioprine	2 (5.26%)	0	2 (9.52%)
Thalidomide	4 (10.53%)	0	4 (19.05%)
Cyclophosphamide	4 (10.53%)	3 (17.65%)	1 (4.76%)
Tacrolimus or ciclosporin	3 (7.89%)	2 (11.76%)	1 (4.76%)
Sirolimus	1 (2.63%)	0	1 (4.76%)
Rituximab	1 (2.63%)	0	1 (4.76%)

*13 (76.47%) patients with renal biopsy and ISN/RPS 2003 class II in 1 (5.88%), III/IV in 4 (23.53%), III/IV+V in 3 (17.65%), V in 5 (29.41%) individuals. The cumulative number of immunosuppressants previously exposed among 17 patients with LN was 4 (IQR 2, 5).

†Disease duration of LN was 2.7 (2, 6.2) years.

‡Duration of diabetes was 0.5 (0.28, 3.61) years.

ALT, alanine transaminase; dsDNA, double-stranded DNA; eGFR, estimated glomerular filtration rate; LN, lupus nephritis; RAASis, renin-angiotensin-aldosterone system inhibitors; SLEDAI, Systemic Lupus Erythematosus Disease Activity Index.

### Safety

A total of 19 AEs (50% of the patients) were reported during the trial, and among them, 7 (18.42% of the patients) were related to disease flares, and 12 (31.58% of patients) were interpreted as being dapagliflozin-related (table 2). Eight (21.05%) patients discontinued dapagliflozin due to AEs, of which four AEs were non-flare-related. Two patients were lost to follow-up, and two patients did not comply with the study protocol. Two serious adverse events (SAEs) were observed, including one case of fungal pneumonia and one case of major disease flare, and both resulted in admission to hospital. The patient with fungal pneumonia had immune thrombocytopenia and received a baseline treatment of prednisone at 25 mg/day, along with azathioprine and thalidomide. The patient presented with fever, productive cough and pulmonary infiltration at 10 weeks after enrolment. Dapagliflozin was terminated, and the microbiology study revealed *Aspergillus fumigatus* infection; the patient was successfully treated with itraconazole.

**Table 2:** Safety

Characteristics, n (%)	SLE (n=38)
Any adverse events (AEs)	19 (50.0%)
AEs led to drug discontinuation	8 (21.05%)
Non-flare AEs which led to drug discontinuation	4 (10.53%)
Serious AEs	2 (5.26%)
Non-flare serious AEs*	1 (2.63%)
Flare	7 (18.42%)
Major flares	3 (7.89%)
Mild-to-moderate flares	4 (10.53%)
Nausea or vomiting	1 (2.63%)
Diarrhoea	1 (2.63%)
Cutaneous pruritus	1 (2.63%)
Asymptomatic pyuria†	5 (13.16%)
Infections	4 (10.53%)
Upper respiratory tract infection	2 (5.26%)
Fungal pneumonia*	1 (2.63%)
Lower urinary tract infection	1 (2.63%)
Death	0
Diabetic ketoacidosis	0
Hypoglycaemia	0

*The patient with fungal pneumonia was also accounted for the serious AE.

†Pyuria was defined as white blood cell count >15/HP in urine analysis and patients were symptom-free. Of which, three were patients without LN and the other two were patients with LN. All without evidence of SLE/LN disease flares.

LN, lupus nephritis; SLE, systemic lupus erythematosus.

Only one (2.63%) patient developed a lower UTI after 16 weeks of treatment with dapagliflozin, which resolved after antibiotic treatment. Of note, five (13.16%) patients developed asymptomatic pyuria (defined as a white blood cell count >15/HP in urinalysis) that was not attributed to SLE disease activity, three of whom were patients without LN and two of whom were patients with LN without renal flares. None of the participants (with or without diabetes) developed major hypoglycaemia or diabetic ketoacidosis or died.

### Efficacy

**Figure 2:**
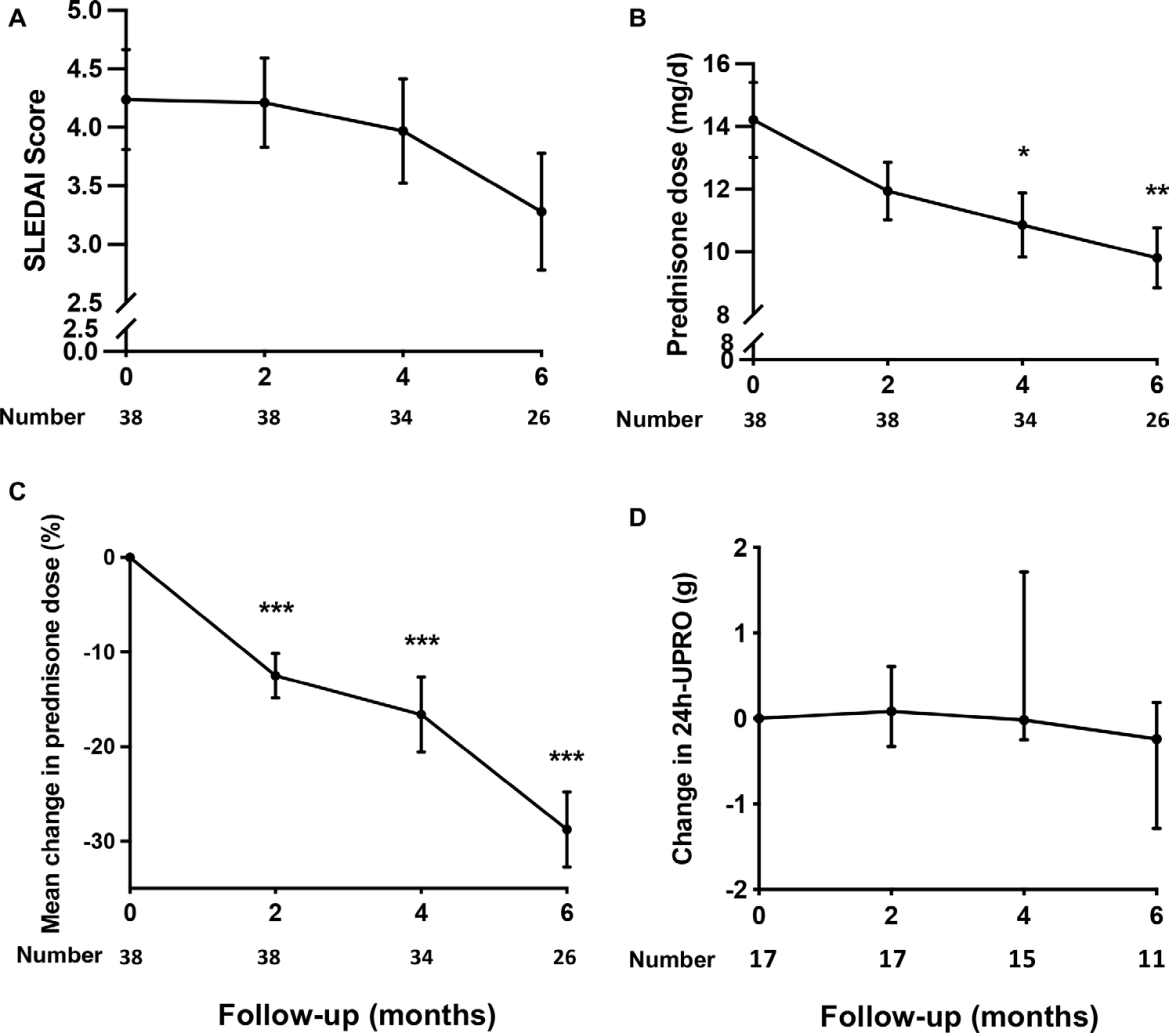
Secondary efficacy outcomes. (A–C) Changes of SLEDAI-2K scores (A), daily dosage (B) and percentage of prednisone (C) from baseline to 6 months. T-bars indicate SE. (D) Changes of median 24-hour urinary protein, T-bars indicate 25th–75th percentile. SLEDAI-2K, Systemic Lupus Erythematosus Disease Activity Index 2000; UPRO, urine protein. *P<0.05, **p<0.001, ***p<0.0001.

At the 0-month, 2-month, 4-month and 6-month follow-ups, the mean SLEDAI score decreased from 4.24 (SD 2.62) at baseline to 3.97 (SD 2.54) at the last visit. The mean change in the SLEDAI score from baseline to the last visit was −0.37 (SD 1.67), which was not statistically significant (table 3 and figure 2A). Similar results were obtained in the subsets of patients with LN (table 3). Seven (18.4%) patients had a disease flare, including three with a major flare and four with a mild-to-moderate flare (table 3). The mean daily prednisone dose was reduced from 14.21 (SD 7.36) mg at baseline to 10.67 (SD 6.14) mg at the last visit, which yielded a 30% reduction (−3.54±5.46 mg/day) (table 3; figure 2B–C). The median 24-hour urinary protein remained unchanged in the 17 patients with active LN, from 1.7 g at baseline (IQR 1.24, 2.95) to 1.7 g (IQR 1.02, 3.43) at the last visit (figure 2D). Except for the prednisone reduction over time, which was compatible with standard-of-care tapering, no signs of decreased disease activity or proteinuria in the patients with LN were observed.

**Table 3:** Secondary and exploratory outcomes at last visit in patients with SLE

Secondary outcomes	SLE (n=38)	P value*	LN (n=17)	P value*	Non-LN (n=21)	P value*
Change in SLEDAI score at last visit	−0.37±1.67	0.24	−0.56±1.81	0.22	0±1.30	1.0
SFI†	7 (18.42%)		4 (23.53%)		3 (14.29%)	
Major flare	3 (7.89%)		1 (5.88%)		2 (9.52%)	
Mild-to-moderate flare	4 (10.53%)		3 (17.65%)		1 (4.76%)	
Change in daily prednisone at last visit, mg	−3.54±5.46	<0.001	−3.60±4.53	0.001	−2.83±6.19	<0.001
Change in 24-hour UPRO at last visit, g			0.14 (−0.34, 0.55)	0.36		
Exploratory outcomes		Nominal p value		Nominal p value		Nominal p value
Change in BMI at last visit, kg/m^2^	−0.50±0.97	0.004	−0.50±0.70	0.01	−0.50±1.16	0.54
Change in SBP at last visit, mm Hg	−3.94±10.57	<0.001	−5.08±9.65	0.04	−2.47±8.87	0.21
Mean SBP over 6 months (within-person SD), mm Hg	119.3 (6.14)	–	117.4 (5.85)	–	121.73 (6.09)	–
Change in DBP at last visit, mm Hg	−0.81±7.44	0.48	−1.63±7.12	0.26	−0.06±6.63	0.89
Change in haemoglobin at last visit, g/L	8.26±15.52	0.003	10.47±20.79	0.06	6.48±11.07	0.11
Change in eGFR at last visit, mL/min/1.73 m^2^	−0.24±12.87	0.90	0.87±16.31	0.73	−1.15±9.58	0.44
6-month eGFR slope, mL/min/1.73 m^2^ per 6 months	0.92±15.54		1.12±17.90		0.75±13.81	

*Calculation based on comparing values at last visit with those at baseline.

†Three patients had major flare including one case of relapse of LN in LN group and two cases of thrombocytopaenia in non-LN group, and four had mild-to-moderate flare including one case of rash in non-LN group and three cases of increased proteinuria in LN group.

BMI, body mass index; DBP, diastolic blood pressure; eGFR, estimated glomerular filtration rate; LN, lupus nephritis; SBP, systolic blood pressure; SFI, Safety of Estrogens in Lupus Erythematosus National Assessment-SLE Disease Activity Flare Index; SLE, systemic lupus erythematosus; SLEDAI, SLE Disease Activity Index; UPRO, urine protein.

**Figure 3:**
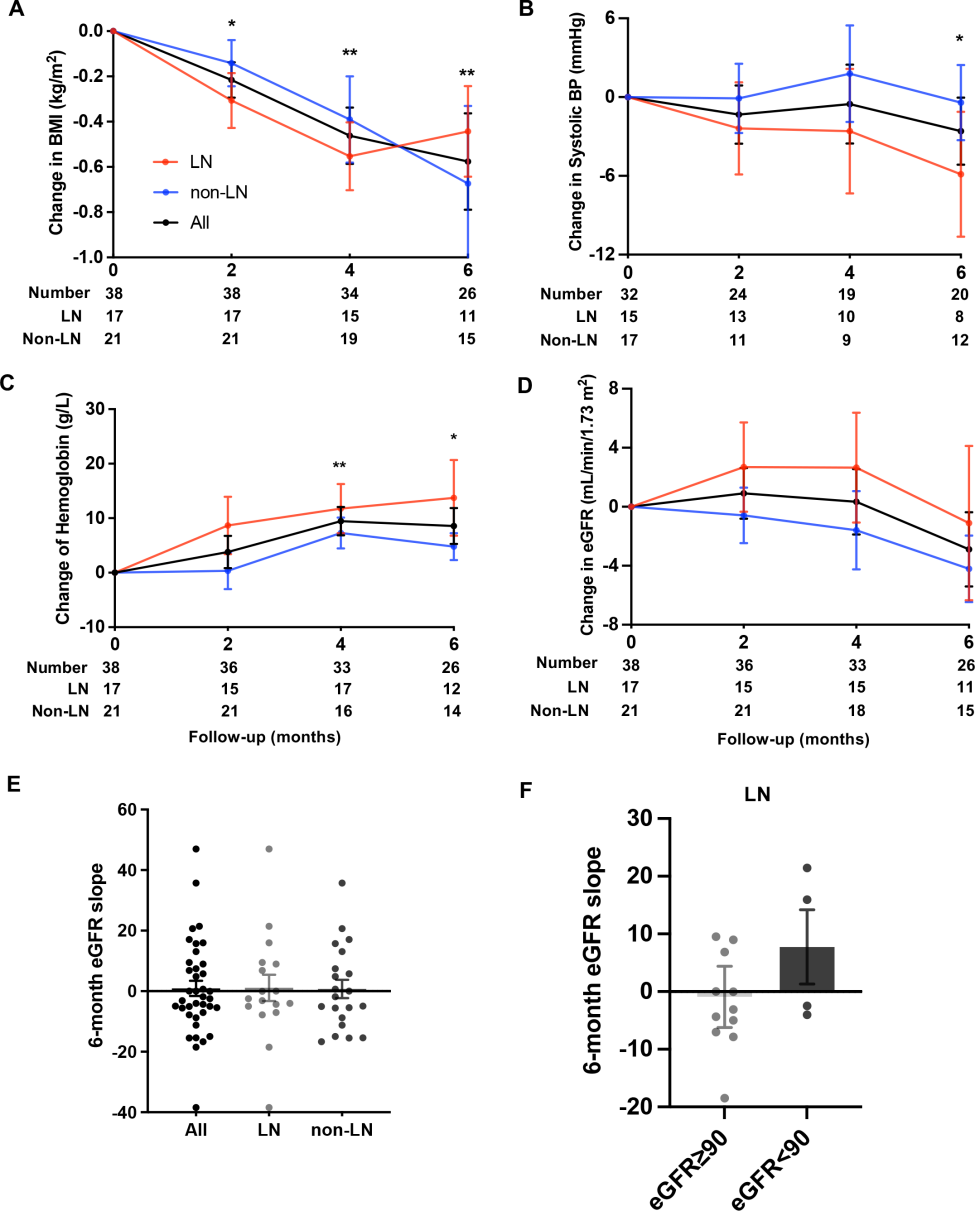
Exploratory outcomes. (A–D) Changes in body mass index (BMI) (A), systolic blood pressure (BP) (B), haemoglobin (C) and estimated glomerular filtration rate (eGFR) (D) from baseline to 6 months. The black lines represent all patients, the red lines represent the LN group and the blue lines represent the non-LN group. Nominal p values were provided only for black lines. *P<0.05, **p<0.001. (E) Mean 6-month eGFR slopes were presented among all patients (n=38), patients with LN (n=17) and patients without LN (n=21). (F) The mean 6-month eGFR slopes in patients with LN with a baseline eGFR value ≥90 (n=13) and <90 mL/min/1.73 m^2^ (n=4) were presented.

Regarding the exploratory outcomes, the mean BMI decreased from 23.78 (3.98) kg/m^2^ at baseline to 23.28 (3.87) kg/m^2^ at the last visit, with a net change of −0.50 (0.97) kg/m^2^ (table 3, figure 3A). The mean systolic BP showed a net change of −3.94 (10.57) mm Hg, declining from 121.32 (15.33) mm Hg at baseline to 115.97 (13.84) mm Hg at the last visit (figure 3B). The diastolic BP remained stable throughout the trial. In addition, an increase in haemoglobin was detected (+8.26 g/L (95% CI 2.99 to 13.54)) (figure 3C). For patients with LN, similar findings for BMI and SBP reductions, along with haemoglobin elevation, were observed (table 3, figure 3A–C). The mean eGFR was stable during the trial (a net change of −0.24 (12.87) mL/min/1.73 m^2^ from the baseline value of 118.40 (27.93) mL/min/1.73 m^2^), and the 6-month eGFR slope was 0.92 (15.54) mL/min/1.73 m^2^ per 6 months (table 3, figure 3D). Furthermore, the 6-month eGFR slopes in patients with and without LN were 1.12 and 0.75 mL/min/1.73 m^2^ per 6 months, respectively (table 3, figure 3E). Intriguingly, the 6-month eGFR slopes in the patients with LN with a baseline eGFR value ≥90 and with a baseline eGFR value <90 mL/min/1.73 m^2^ were −0.92 and 7.73 mL/min/1.73 m^2^ per 6 months, respectively (figure 3F). Thus, there was a signal of improvement of the eGFR slope among patients with LN who had a baseline eGFR of <90 mL/min/1.73 m^2^.

With respect to the effects of dapagliflozin on diabetes, five patients with SLE with a 0.5 (0.28–3.61) year duration of diabetes were analysed. The mean baseline HbA1c and FBG levels were 9.9 (4.6) % and 8.3 (2.3) mmol/L, respectively. As expected, they experienced better HbA1c and FBG control during the trial (6.7 (0.63) % and 6.12 (0.98) mmol/L at the last visit, respectively, online supplemental table S1)). Due to the small number of patients, further subgroup analysis was not carried out.

10.1136/rmdopen-2022-002686.supp1 Supplementary data

## Discussion

SLE is a chronic systemic autoimmune disease, and the kidney is commonly involved. Approximately 10%–30% of patients with LN will progress to end-stage renal disease within 15 years.^29^ In addition, a high risk of cardiovascular events is one of the leading causes of mortality in patients with long-term SLE.^30 31^ The unique cardiac/renal protective effects of SGLT2is in patients with CKD offer an attractive opportunity in SLE/LN management.^10 11 15^ To the best of our knowledge, this is the first trial to explore the safety and efficacy of dapagliflozin add-on therapy to the background of the standard of care in patients with SLE. The trial provides evidence that up to 6 months of dapagliflozin exposure had an acceptable safety profile in adult patients with SLE; however, the potential cardiac/renal protective effects are still inconclusive.

An AE with special interest is a UTI. SGLT2is, such as dapagliflozin, increase the availability of glucose in the urinary tract; therefore, UTI is a common side effect.^26^ Given that patients with SLE are susceptible to different infections, UTI becomes a concern. However, UTI was observed in only one (2.63%) patient in our trial, which was not higher than that in non-SLE large-scale clinical trials.^14 32^ The UTI frequency was also compatible with the AE report in the placebo arm of our previous SLE RCT.^5^ It is noteworthy that five patients (13.16%), including three patients without LN and two patients with LN, had new-onset asymptomatic pyuria while receiving dapagliflozin treatment. The clinical meaning of this finding was undetermined and requires further long-term observation. Invasive fungal infection was recorded in one patient. No increased risk of fungal pneumonia relevant to SGLT2is has been reported. A population-based cohort study provided reassuring evidence that SGLT2is were associated with a decreased rate of community-acquired pneumonia among patients with type 2 diabetes.^33^ Nevertheless, further surveillance of this SAE should not be overlooked. The overall rate of drug-related AEs resulting in drug discontinuation in this trial was ~10%. Other AEs of interest, such as hypoglycaemia, diabetic ketoacidosis, fracture, volume depletion, acute kidney injury or malignancy, were not observed. Our current data preliminarily confirmed an acceptable safety profile of dapagliflozin in Chinese adult patients with SLE.

In this study, the mean baseline SLEDAI score of 4 suggested that these participants had only low-grade disease activity. No clinically meaningful changes in the SLEDAI score over time were observed. In addition, an 18% 6-month cumulative flare rate was observed in this trial, which is in line with the frequency of flares in the control arm with the standard of care (~40% annual flare rate) among a similar SLE population with a baseline SLEDAI score of 4 from our previous SLE trial.^5 6^ Although the prednisone dosage was reduced by ~30% from baseline to the last visit, this was also consistent with the standard tapering protocol in practice. Recently, a case series of five active patients with LN demonstrated a promising proteinuria reduction (~50% reduction within 8 weeks) with an SGLT2i (empaglifozin) add-on to stable immunosuppressive treatment.^28^ Unfortunately, for patients with LN (n=17) in our trial, dapagliflozin add-on therapy did not help reduce the proteinuria level. One explanation is that <50% of our patients with LN were on RAASis, which has been postulated to be required for SGLT2is to enhance their action.^15 34^ The other possible reason might be attributed to the fact that the participants had a relatively long LN duration (2.7 years) and were resistant to multiple (average of 4) previous immunosuppressants. Altogether, the secondary efficacy end points of SGLT2is were not substantial.

Nevertheless, the exploratory end points suggested potential cardiac/renal protective effects of dapagliflozin in patients with SLE. Cardiovascular risk factors such as BMI and BP improved during follow-up. The mode of action of SGLT2is is to increase sodium delivery to the macula densa, which reduces tubuloglomerular feedback and decreases intraglomerular pressure. It has been reported that SGLT2is can reduce tubular work and oxygen requirements, thereby reducing the damage associated with hypoxic tubular cells and enhancing renal erythropoietin production.^35^ Our data further revealed an increase in haemoglobin levels, an effect that has been captured in patients with or without diabetes with CKD treated with SGLT2is.^14 36^ Possibly via SGLT2i-stimulated erythropoiesis,^15^ the increase in haemoglobin might in turn improve oxygen delivery to vital organs.^37^ Our data indicated a stable 6-month eGFR among our patients with SLE who received dapagliflozin treatment. Interestingly, we observed a 6-month eGFR slope elevation among patients with LN with a baseline eGFR level <90 mL/min/1.73 m^2^. In other words, SGLT2is might display a nephroprotective effect in a more pronounced way among patients with LN with renal dysfunction (CKD grade 2 or higher).

Importantly, SGLT2is or dapagliflozin block mTOR activation in macrophages as well as in podocytes.^23 38^ It is well established that mTOR plays a key role in adaptive immune system activation, proinflammatory lineage development^39^ and podocyte and endothelial cell dysfunction,^40^ which underscores the implication of mTOR blockade in patients with SLE.^41 42^ SGLT2is or dapagliflozin might act, at least partially via mTOR inhibition, as adjuvant immune modulatory treatments for SLE along with their renal/cardiac protective properties. The combination of the potential beneficial effects of SGLT2is is calling for a phase IIb trial with proper efficacy end points, such as eGFR slope, to be conducted among patients with LN who have impaired renal function, for example, an eGFR level of 30~90 mL/min/1.73 m^2^. The target LN population subject to SGLT2i add-on therapy would be preferred after induction therapy with a 1-year to 2-year study.

The major limitation of this study is that this was a single-centre, open-label, uncontrolled small trial. With the aim of addressing the safety issue of up to 6 months of exposure to dapagliflozin in patients with SLE, any overinterpretation or inappropriate extrapolation of the outcome should be avoided, especially for efficacy analyses.

In conclusion, dapagliflozin had an acceptable safety profile in adult patients with SLE. No distinct effects in terms of reducing disease activity or proteinuria among patients with LN were detected. Its possible renal/cardiac protective effects and long-term safety issues in patients with SLE, patients with LN in particular, await larger-scale, placebo-controlled trials.

## Data Availability

Data are available on reasonable request. Data generated and/or analysed during the current study are available from the corresponding author on reasonable request.
