# Supplementary material for: Safety and efficacy of the SGLT2 inhibitor dapagliflozin in patients with systemic lupus erythematosus: a phase I/II trial
Source: RMD Open. 2022 Oct 26;8(2):e002686. doi: 10.1136/rmdopen-2022-002686 (PMC9615980; doi:10.1136/rmdopen-2022-002686)
Supplement: Supplementary data [file rmdopen-2022-002686supp001.pdf]

**Table S1 Main effects of Dapagliflozin in SLE patients with diabetes**

| N | 1 | 2 | 3 | 4 | 5 |
|---|---|---|---|---|---|
| Age/sex | 60/F | 33/F | 45/F | 32/F | 55/F |
| Antidiabetic drugs | Glipizide | Acarbose, IRI | IRI | Acarbose, IRI | IRI |
| eGFR baseline | 102.49 | 142.6 | 77.0 | 134.1 | 60.96 |
| eGFR 6 months | 96.94 | 104.7 | 96.3 | 143.7 | 73.70 |
| Proteinuria at baseline (g/24h) | - | 2.89 | 1.89 | - | 1.7 |
| Proteinuria at 6 months (g/24h) | - | 1.45 | 2.08 | - | 7.05 |
| HbAlc baseline (%) | 6.8 | 17.9 | 9.4 | 7.6 | 7.6 |
| HbAlc 6 months (%) | 6.9 | 5.6 | 7.2 | 6.9 | 6.9 |
| FBG baseline (mmol/L) | 5.71 | 9.68 | 6 | 10.2 | 10.1 |
| FBG 6 months (mmol/L) | 5.84 | 4.82 | 5.91 | 6.97 | 7.27 |
| SBP baseline (mmHg) | 115/71 | 114 / 87 | 120/80 | 124/101 | - |
| SBP 6 months (mmHg) | 122/76 | 142/98 | 129/90 | 118/90 | - |
| BMI baseline ($kg/m^2$) | 25.36 | 18.29 | 24.35 | 21.26 | 21.48 |
| BMI 6 months ($kg/m^2$) | 21.91 | 18.65 | 23.31 | 19.84 | 20.31 |

IRI, insulin; FBG, fasting blood-glucose; HbAlc, glycosylated hemoglobin; SBP, systolic blood pressure; BMI, body mass index.
